# Effects of Black Adzuki Bean (*Vigna angularis*) Extract on Proliferation and Differentiation of 3T3-L1 Preadipocytesinto Mature Adipocytes

**DOI:** 10.3390/nu7010277

**Published:** 2015-01-06

**Authors:** Mina Kim, Jeong-Eun Park, Seok-Bo Song, Youn-Soo Cha

**Affiliations:** 1 Department of Food Science and Human Nutrition, Chonbuk National University, 664-14 Duckjin-dong, Jeonju, Jeonbuk 561-756, Korea; E-Mails: als@jbnu.ac.kr (M.K.); aich21@jbnu.ac.kr (J.-E.P.); 2 Department of Functional Crop, National Institute of Crop Science, Rural Development Administration, Miryang, Gyeongnam 627-803, Korea; E-Mail: songsb1254@korea.kr

**Keywords:** adzuki bean, *Vigna angularis*, 3T3-L1, obesity, adipocyte, black bean

## Abstract

The aim of this work was to investigate the effects of black adzuki bean (BAB) extract on adipocytes, and to elucidate the cellular mechanisms. In order to examine the proliferation of preadipocytes and differentiating adipocytes, cell viability and DNA content were measured over a period of time. Lipid accumulation during cell differentiation and the molecular mechanisms underlying the effects of BAB on the transcriptional factors involved, with their anti-adipogenic effects, were also identified. We observed that BAB exhibits anti-adipogenic effects through the inhibition of proliferation, thereby lowering mRNA expression of C/EBPβ and suppressing adipogenesis during the early stage of differentiation. This, in turn, resulted in a reduction of TG accumulation in a dose- and time-dependent manner. Treating the cells with BAB not only suppressed the adipogenesis-associated key transcription factors PPARγ and C/EBPα but also significantly decreased the mRNA expression of GLUT4, FABP4, LPL and adiponectin. The expression of lipolytic genes like ATGL and HSL were higher in the treatment group than in the control. Overall, the black adzuki bean extract demonstrated an anti-adipogenic property, which makes it a potential dietary supplement for attenuation of obesity.

## 1. Introduction

A constant positive energy balance can lead to excessive fat accumulation in white adipose tissues. Excessive body fat, especially higher visceral adiposity, is associated with an increased risk in the development of numerous adverse health conditions including diabetes and cardiovascular disease [1,2,3]. Obesity is induced by an increase in adipose tissue mass, which results from the multiplication of fat cells followed by adipogenesis and increased deposition of cytoplasmic triglycerides [4]. Adipogenesis is a process of formation of new adipocytes from preadipocyte precursors, and an understanding of this process is important for controlling obesity [5]. Adipogenesis is a complex process called thatis sequentially regulated by several transcription factors such as peroxisome proliferator-activated receptor gamma (PPARγ) and CCAAT/enhancer binding proteins (C/EBPβ, C/EBPδ and C/EBPα). Differentiation of 3T3-L1 cells into adipocytes involves a highly orchestrated series of events including clonal expansion, growth arrest, and terminal differentiation [6].

However, adipose tissue mass can be decreased by either inhibiting adipogenesis or inducing apoptosis of adipocytes. Accordingly, understanding the mechanisms by which a particular nutrient affects adipocyte differentiation may help in developing strategies to prevent obesity and its associated diseases [7].

A large body of literature indicates that substantial progress has been made in developing plant-based foods that may prevent obesity and contribute beneficial health effects without any known harmful side effects [7]. Natural products capable of inhibiting adipogenesis and inducing apoptosis of adipocytes will have great potential for treating and preventing obesity [1].

The adzuki bean (*Vigna angularis*), is one of the most important food crops and folk medicines in South Korea, China, Japan, and Taiwan [8]. The bean comes in various colors, such as red, black, yellow, and white [8,9], resulting from the presence of different pigments that are known to include polyphenols such as proanthocyanidins and quercetin, which have therefore received considerable attention owing to their well-documented antioxidant activities [9,10,11]. Studies have also reported that their hypocholesterolemic [12,13] and hypoglycemic effects [14], along with potent antioxidant activity, help lower blood pressure, vascular oxidative stress, and inflammation [8,9,10]. Despite continuous interest in the adzuki bean as a rich source of therapeutic and/or preventive compounds against many diseases, few studies have evaluated the direct effects of adzuki beans on adipocyte differentiation.

We therefore investigated and found that black adzuki bean (BAB) mediated suppression of adipogenesis via impairing the proliferation and thus differentiation of adipocytes. Considering that the biological activities of a combination of phytochemicals have synergistic effects [15,16,17], we investigated the effects of the extract from BAB on adipocyte function instead of a single compound.

## 2. Materials and Methods

### 2.1. Sample Preparation and Reagents

Black adzuki beans (BAB) were obtained from the National Institute of Crop Science, Gyeongsangbuk-do, Korea in 2013. Red adzuki beans (RAB) and black soybeans (BB) were purchased from Nonghyup’s Hanaro Club. The bean extract was prepared by adding 80% ethanol to the ground beans, which were then concentrated by evaporating the ethanol using a rotary vacuum evaporator (N-1110, Eyela, Tokyo, Japan), freeze-dried, and stored at −20 °C for further use. The concentration of each extract was expressed based on the dry mass of the extract. The composition analyses according to food ingredients test methods of the Korean Food Standards Codex (KFSC, 2011) were performed by the Dasan Institute of Life & Science Co., LTD, Gwangju, Korea. The composition per 100 g of freeze-dried powder samples of the beans was as follows: 76.7 g carbohydrate, 0.7 g protein, 8 g lipid, 3.3 g ash, 0.7 g fiber, 0.5 g total flavonoids in BAB; 22 g carbohydrate, 44 g protein, 18 g lipid, 9 g ash, and 0.8 g total flavonoids in RAB; and 70 g carbohydrate, 3 g protein, 9 g lipid, 6 g ash, 1 g fiber, and 0.3 g total flavonoids in BB.

1,1-Diphenyl-2-picrylhydrazyl (DPPH), 2,2’-azino-bis-(3-ethylbenzthiazoline-6-sulfonic acid) diammonium salt (ABTS), potassium persulfate, trolox, ascorbic acid, 3-isobutyl-1-methyl-xanthine (IBMX), dexamethasone (DEXA), insulin, trypsin-EDTA, and Oil Red O were purchased from Sigma-Aldrich Co. (St. Louis, MO, USA). Dulbecco’s modified Eagle’s medium (DMEM) was supplied by Lonza (Walkersville, MD, USA), penicillin-streptomycin was obtained from Hycolone (Logan, UT, USA), and bovine serum (BS) and fetal bovine serum (FBS) were bought from Gibco (Grand Island, NY, USA). EZ-Cytox (#EZ-3000) was purchased from Daeil Lab Service (Seoul, Korea). Labeling with BrdU was done using the Cell Proliferation ELISA BrdU colorimetric kit (Roche, Mannheim, Germany). A triglyceride quantification kit (#K622-100) was supplied by Biovision Inc. (Mountain View, CA, USA). Trizol reagent was obtained from Invitrogen (Carlsbad, CA, USA).

### 2.2. Determination of Antioxidant Activity

The total radical scavenging capacity was determined and compared with ascorbic acid and trolox by using the DPPH and ABTS^+^ radical scavenging methods. The antioxidant capacity of the bean extracts on DPPH radicals was determined by the method of Blois [18] with suitable modifications. Briefly, 0.15 mM solution of DPPH was prepared in ethanol and 0.16 mL of this solution was added to 0.04 mL of bean extract in ethanol at different concentrations. The mixture was kept at room temperature for 30 min, and then the absorbance was recorded at 517 nm. The control was treated with 95% ethanol. The ABTS^+^ was produced by reacting 7.4 mM ABTS with 2.6 mM potassium persulfate (K_2_S_2_O_8_) and storing it in a dark at room temperature for 12 h. The ABTS^+^ solution was diluted to give an absorbance of 1.2–1.5 at 734 nm. Then 0.95 mL of ABTS^+^ solution was added to 0.05 mL of bean extract in ethanol at different concentrations. The absorbance was recorded 30 min after mixing and the concentration of bean extract providing 50% inhibition (IC_50_) was estimated from a graph plotting percent inhibition against bean extract concentration (mg/mL).

### 2.3. Cell Culture and Differentiation

The 3T3-L1 mouse preadipocyte cell lines (CL-173) were obtained from American Type Culture Collection (ATCC, Manassas, VA, USA) and cultured in DMEM containing 10% BS and 100 U/mL penicillin-streptomycin at 37 °C in a humidified 5% CO_2_ incubator. 3T3-L1 preadipocytes were seeded into 6-well plates and were differentiated into mature adipocytes. To induce differentiation, two days after post-confluency, the cells were stimulated with induction medium containing DMEM with 10% FBS and MDI solution (0.5 mM IBMX, 1 μM DEXA, and 10 μg/mL insulin) for 3 days. After the cells were maintained in 10% FBS and 10 μg/mL of insulin in DMEM for another 8 days, 90% of the cells had transformed into mature adipocytes, with formation of intracellular lipid droplets. The medium, along with various concentrations of BAB, was replenished every other day during the differentiation (full period; 1st to 11th day) or the differentiating cells were treated with BAB in different time periods; for the full period, cells were cultured in the presence of BAB for 11 days (full period; 1st to 11th day), for the early period cells were treated with BAB for the first 3 days (early period; 1st to 3rd day), and for the terminal period the cells were treated with BAB for the last 3 days of the differentiation period (terminal period; 9th to 11th day).

### 2.4. MTT Assay

The effect of BAB on cell proliferation was assessed with EZ-Cytox reagent [19]. 3T3-L1 preadipocytes were seeded in 96-well plates (2500 cells/well) and allowed to adhere overnight. After discarding the medium, a culture medium containing BAB (0.5 to 4 mg/mL) was added to each well and the cells were incubated for 24 to 72 h; untreated cells were used as controls. After completion of the time period, the medium was replaced with a medium containing EZ-Cytox solution (0.01 mL/well) and incubated at 37 °C for an additional 2 h. Absorbance was measured at 450 nm using a microplate reader (MRX II, Dynex Technologies, Chantilly, VA, USA). Cell proliferation (%) was calculated by the following equation:

(absorbance of the sample/mean absorbance of the control) × 100 (1)

### 2.5. Bromodeoxyuridine Incorporation Assays

3T3-L1 preadipocytes were grown up to 2 days postconfluency in a 96-well plate, and then induced to differentiate with BAB (1 mg/mL, 2 mg/mL) for 24 h as described above. At 8, 12, and 24 h after stimulation with MDI in the presence or absence of BAB, BrdU solution (10 μM) was added to the culture medium. After BrdU labeling for 2 h, the medium was removed and BrdU incorporation into the DNA was measured using the Cell Proliferation ELISA BrdU colorimetric kit (Roche).

### 2.6. Triglyceride Assay and Oil Red O Staining

For TG quantification, the harvested cells were washed twice with PBS, homogenized in 5% Triton X-100, and then assayed using triglyceride quantification at 580 nm.

When preadipocytes are differentiated into adipocytes, they accumulate intracellular lipid droplets, which can be stained with Oil Red O [20,21]. The lipid droplets were evident in fully mature differentiated adipocyte control compared with those of preadipocyte control. Briefly, after being fixed in 10% formalin for 60 min, the cells were washed twice with PBS and stained with filtered Oil Red O solution (1.8 mg/mL Oil Red O and 60% (v/v) isopropanol in distilled water) for 60 min. The cells were then thoroughly washed with distilled water before being photographed under an optical microscope (CKX41, Olympus, Tokyo, Japan).

### 2.7. Quantitative Real-Time Polymerase Chain Reaction Analysis

Total RNA was extracted using a Trizol reagent and the concentration was measured spectrophotometrically at 260 and 280 nm. The extracted RNA was reverse transcribed into cDNA using a high-capacity cDNA reverse transcription kit (Applied Biosystems, Foster City, CA, USA). Then the RNA expression level was quantified by quantitative real-time PCR using SYBR Green PCR Master Mix (Applied Biosystems, Woolston, Warrington, UK) and the 7500 Real Time PCR system (Applied Biosystems, Foster City, CA, USA) according to the manufacturer’s protocol. Gene-specific primers used are as follows: C/EBPβ F: 5′-GTTTCGGGACTTGATGCAATC-3′ and R: 5′-AACAACCCCGCAGGAACAT-3′; C/EBPδ F: 5′-GATCTGCACGGCCTGTTGTA-3′ and R: 5′-CTCCACTGCCCACCTGTCA-3′; C/EBPα F: 5′-GTGTGCACGTCTATGCTAAACCA-3′ and R: 5′- GCCGTTAGTGAAGAGTCTCAGTTT-3′; PPARγ F: 5′- GTGCCAGTTTCGATCCGTAGA-3′ and R: 5′-GGCCAGCATCGTGTAGATGA-3′; glucose transporter4 (GLUT4) F: 5′-GATTCTGCTGCCCTTCTGTC-3′and R: 5′- ATTGGACGCTCTCTCTCCAA-3′; adiponectin F: 5′-GCACTGGCAAGTTCTACTGCAA-3′ and R: 5′-GTAGGTGAAGAGAACGGCCTTGT-3′; lipoprotein lipase (LPL) F: 5′-ACTCGCTCTCAGATGCCCTA-3′ and R: 5′-TTGTGTTGCTTGCCATTCTC-3′; fatty acid binding protein4 (FABP4) F: 5′-ATGATCATCAGCGTAAATGG-3′ and R: 5′-GCCTTTCATAACACATTCCA-3′; adipose triglyceride lipase (ATGL) F: 5′-AACACCAGCATCCAGTTCAA-3′ and R: 5′-GGTTCAGTAGGCCATTCCTC-3′; hormone sensitive lipase (HSL) F: 5′-ACCGAGACAGGCCTCAGTGTG-3′ and R: 5′-GAATCGGCCACCGGTAAAGAG-3′; and β-actin F: 5′-AGCCTTCCTTCTTGGGTATGG-3′ and R: 5′-CACTTGCGGTGCACGATGGAG-3′. Relative quantification of gene expression with real-time PCR data was calculated relative to β-actin.

### 2.8. Statistical Analysis

Data from individual experiments are expressed as the means ± SD. The data were analyzed by one-way ANOVA using the SPSS 12.0 program and the differences between the means were assessed using Duncan’s multiple range test. To examine the antioxidant activity and mRNA expression (C/EBPβ and C/EBPδ), an independent *t*-test was used. Statistical significance was considered at *p* < 0.05.

## 3. Results

### 3.1. Antioxidant Activity of Bean Extracts

We evaluated the antioxidant activity of 20 different kinds of adzuki bean ethanol extract (Supplementary Table S1) and BAB exhibited effective antioxidant activity compared to the rest of the adzuki beans. The IC_50_ values of BAB for scavenging DPPH (0.98 mg/mL) and ABTS (2.00 mg/mL) radicals were significantly lower compared to RAB and BB (Table 1 and Supplementary Figure S1).

**Table 1:** IC_50_ values of the bean extracts, as determined by DPPH and ABTS^+^ assay.

IC_50_(mg/mL)	Ascorbic Acid	Trolox	BAB	RAB	BB
DPPH	0.06 ± 0.01 ^d^	0.05 ± 0.02 ^d^	0.98 ± 0.01 ^c^	1.34 ± 0.02 ^b^	9.59 ± 0.14 ^a^
ABTS	0.10 ± 0.02 ^d^	0.19 ± 0.01 ^d^	2.00 ± 0.07 ^c^	2.53 ± 0.04 ^b^	7.35 ± 0.09 ^a^

Data are expressed as means ± SD. A different letter in a row indicates a significant difference among groups, according to ANOVA with Duncan’s multiple range test (*p* < 0.05). BAB: black adzuki bean; RAB: red adzuki bean; BB: black soybean.

### 3.2. Effects of Black Adzuki Bean on Proliferation

**Figure 1:**
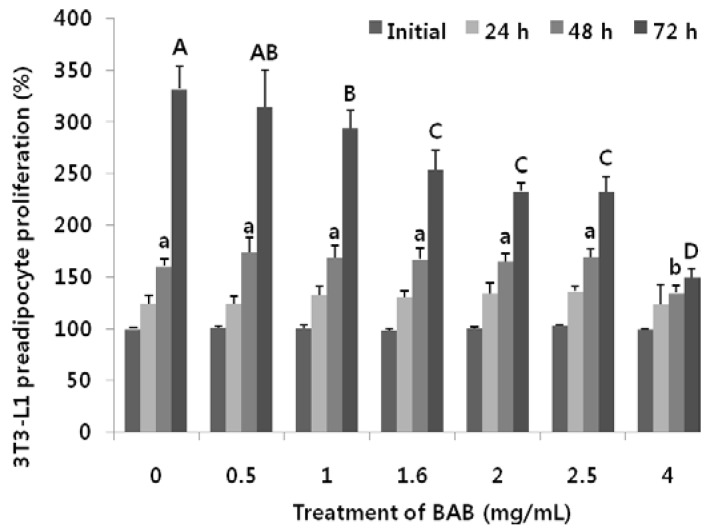
Effects of black adzuki bean on cell viability and preadipocyte proliferation rate of 3T3-L1. 3T3-L1 preadiocytes were exposed to different concentrations (0.5 to 4 mg/mL) of BAB extract for 24–72 h. Cell viability was measured by MTT assays and calculated as relative values *vs.* non-treatment controls (0 mg/mL) cells at the initial time point. Data are expressed as means ± SD and a different small letter (48 h) or capital mark letter (72 h) indicates a significant difference among groups, according to ANOVA with Duncan’s multiple range test (*p* < 0.05).

Prior to determining the effect of BAB on lipid accumulation associated with adipocyte differentiation in 3T3-L1 cells, we confirmed the inhibition of proliferation rate through BAB treatment (Figure 1). The preadipocytes were treated with BAB at increasing concentrations from 0.5 to 4 mg/mL for 24, 48, and 72 h, with untreated cells as non-treatment control. At 0 h (initial) and 24 h, there were no significant differences between and within the groups. Non-treatment controls exhibited normal growth. BAB was not cytotoxic to the preadipocytes between the ranges of 0.5 to 2.5 mg/mL, compared to non-treatment controls at 24–48 h. At 72 h, the inhibition of cell proliferation rate was observed to be 12% (1 mg/mL) and 30% (2 mg/mL), compared to non-treatment controls. At this range, we considered that preadipocyte proliferation might be inhibited in a dose- and time-dependent manner.

### 3.3. Antiadipogenic Function of Black Adzuki Bean in the Early Phase of Adipogenesis

**Figure 2:**
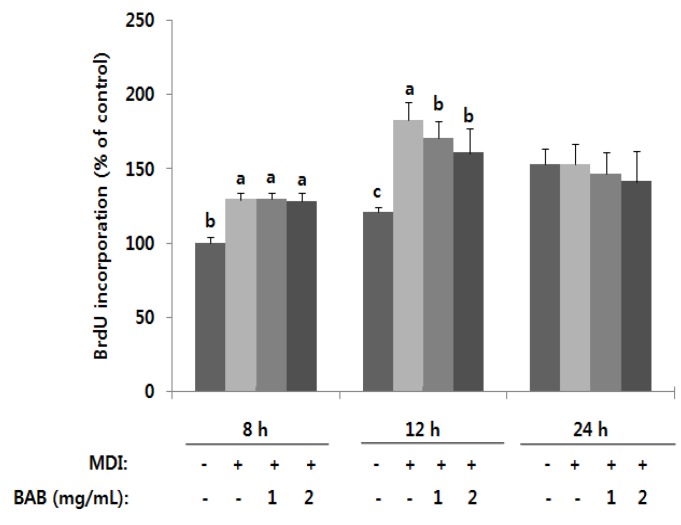
Effect of black adzuki bean on DNA synthesis. Two days postconfluent, preadipocytes were initiated to differentiate with MDI in the presence or absence of black adzuki bean extract. These cells were harvested at the indicated time points (8, 12, and 24 h) after initiating differentiation and were subjected to cell S phase by BrdU incorporation assay. Data are expressed as means ± SD and a different letter indicates a significant difference among groups, according to ANOVA with Duncan’s multiple range test (*p* < 0.05).

We examined the effect of BAB on inhibition of S phase entry during mitotic clonal expansion (MCE); the 3T3-L1 cells were treated with BAB at the time of induction of differentiation. MCE is followed by a critical adipocyte-specific event, necessary for the progression of terminal differentiation [22]. BAB inhibits the entry of adipocytes into S phase, as indicated by the inhibition of BrdU incorporation into DNA (Figure 2). BrdU labeling identifies labeled nuclei in S phase. After 12 h of induction, MDI-treated cells were labeled with BrdU; in contrast, BAB-treated cells showed a significantly decreased level of labeling. These results indicated that the antiadipogenic effect of BAB is attributed to the early phase of adipogenesis.

**Figure 3:**
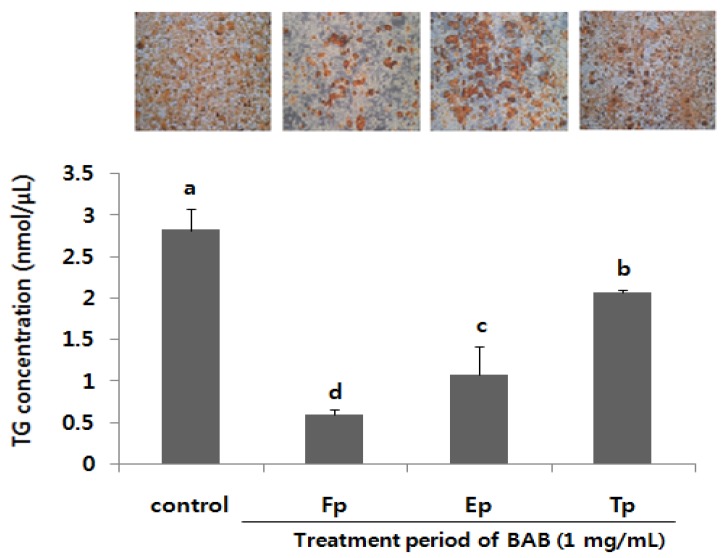
Lipid accumulation of differentiated adipocytes treated with or without black adzuki bean for the indicated period. Cells were measured for TG concentration and stained with Oil Red O and observed under the microscope. Data are expressed as means ± SD and a different small letter indicates a significant difference among groups, according to ANOVA with Duncan’s multiple range test (*p* < 0.05). Fp: full period; Ep: early period; Tp: terminal period; BAB: black adzuki bean.

3T3-L1 adipocyte differentiation initiated by exposure to the adipogenic cocktail consists of three distinct stages, including the early stage (Days 0–2), the post-mitotic intermediate stage (Days 3–4) and the terminal stage (after Day 4) [23,24]. In order to elucidate the underlying concept when BAB inhibited adipocyte differentiation, we attempted to identify the different periods (Fp, full period; Ep, early period; Tp, terminal period) of adipocyte differentiation that are specifically affected by BAB treatment (1 mg/mL). After 11 days of differentiation, the levels of adipocyte differentiation of these cells were determined by quantitative and qualitative analysis (Figure 3). The 3T3-L1 cells treated with BAB for 11 days (Fp) showed an 80% inhibition of adipocyte differentiation. Similarly, the Ep group exhibited significantly (62%) lower levels of intracellular lipid droplets. However, the Tp group exhibited only a slight inhibition of adipocyte differentiation compared with non-treatment control cells. These results suggest that inhibition of adipocyte differentiation by BAB begins in the early stage of differentiation itself.

**Figure 4:**
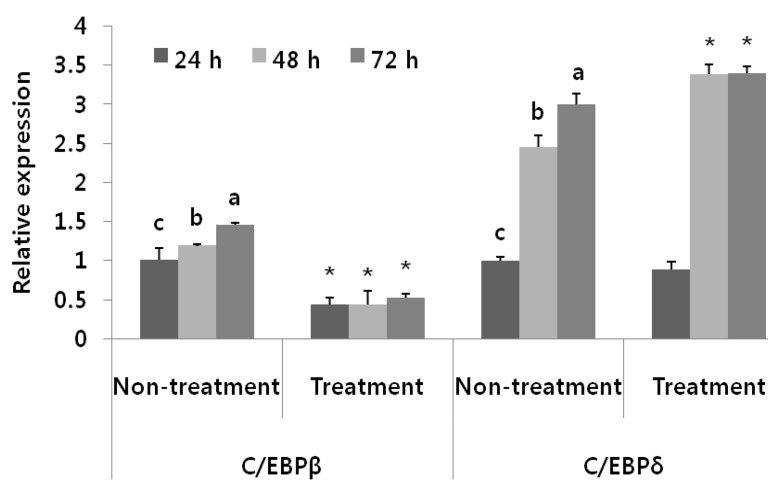
Effects of black adzuki bean on mRNA expression of the early transcription factors expressed during differentiation for the indicated periods. C/EBPβ and C/EBPδ mRNA expression was measured by qRT-PCR. The mRNA expression was normalized using β-actin and shown by relative expression compared to the untreated control group at 24 h. Data are expressed as means ± SD with different letters indicating a significant difference among the non-treatment control group, according to ANOVA with Duncan’s multiple range test (*p* < 0.05) and treatment group compared with untreated (* *p* < 0.05) by Independent *t*-test.

Due to the effect of BAB on lipid deposition in the early stage, we therefore examined the effect of BAB on mRNA levels of the early adipogenic transcription factors C/EBPβ and C/EBPδ in the presence or absence of BAB (1 mg/mL) at different time points during differentiating Days 1–3 (Figure 4). Within 48 h after induction of adipocyte differentiation, the cells completed this coordinated activation of MCE and early adipogenic transcriptional program, and began to express late markers of adipocyte differentiation [23,24]. C/EBPβ is induced very early and plays a crucial role in initiating the differentiation program. The mRNA expression of C/EBPβ in BAB treatment was significantly decreased by approximately 56%–64% compared with the untreated controls.

### 3.4. Adipogenic Levels and Lipid Metabolic Gene Profiles Were Highly Modulated Due to Black Adzuki Bean Extract Treatment

**Figure 5:**
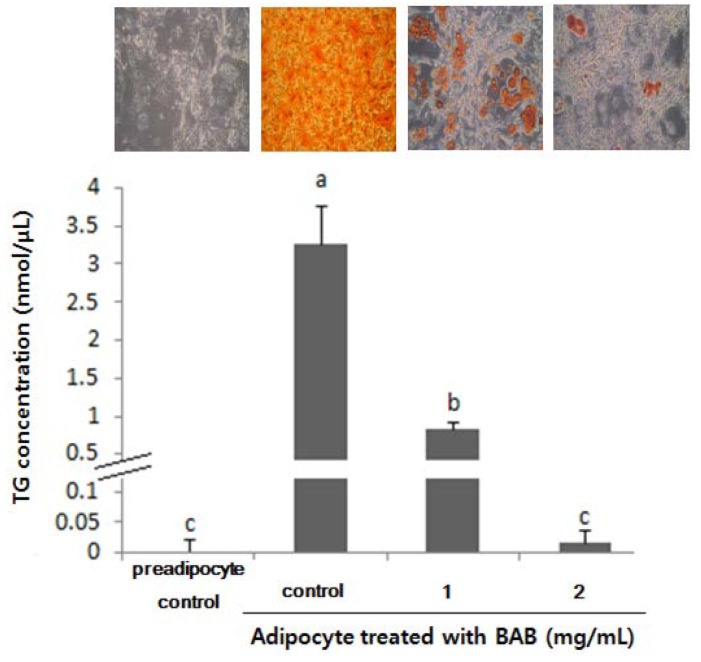
Effects of black adzuki bean extract on lipid accumulation in 3T3-L1 cells.3T3-L1 preadipocyte and differentiated adipocytes treated with black adzuki bean extract (0 to 2 mg/mL) were incubated for 11 days. Cells were stained with Oil Red O and observed under the microscope. Data are expressed as means ± SD and a different letter indicates a significant difference among cells treated with black adzuki bean, according to ANOVA with Duncan’s multiple range test (*p* < 0.05).

We checked the TG accumulation levels at 11 days post induction of full differentiation. The effects of different concentrations of BAB on TG accumulation are shown in Figure 5. TG concentrations in adipocyte cells treated with BAB were significantly lower (1 mg/mL: 0.83 ± 0.10 nmol/μL, 2 mg/mL: 0.02 ± 0.02 nmol/μL) as compared to the adipocyte control (non-treatment: 3.26 ± 0.50 nmol/μL). Treatment with BAB resulted in a dose-dependent decrease in TG accumulation, compared to the adipocyte control. The addition of BAB during adipogenesis almost completely abolished lipid droplet formation and markedly reduced the TG accumulation.

**Figure 6:**
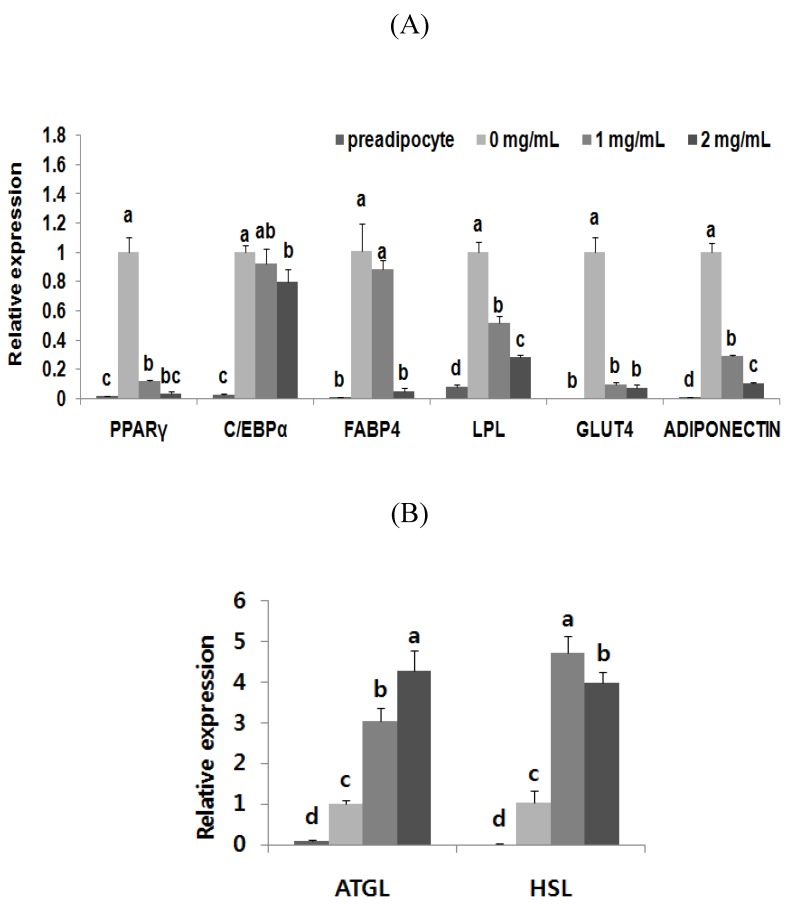
Effects of black adzuki bean on mRNA expression of adipogenesis and lipid metabolism-related genes in 3T3-L1 cells. 3T3-L1 preadipocyte and differentiated adipocytes were incubated for 11 days in media containing different concentration of black adzuki bean extract and then in (**A**) key transcription factors PPARγ and C/EBPα along with FABP4, LPL, GLUT4, and Adiponectin. (**B**) ATGL and HSL mRNA expression were measured by qRT-PCR. The mRNA expression was normalized using β-actin and shown by relative expression of adipocytes in control group (0 mg/mL). Data are expressed as means ± SD with different letters indicating a significant difference among cells treated with black adzuki bean, according to ANOVA with Duncan’s multiple range test (*p* < 0.05).

After the expression of C/EBPβ, during the induction of adipocyte differentiation, the subsequent activation of the transcriptional cascade is required for terminal adipocyte differentiation. The mRNA expression levels of the genes measured—PPARγ and C/EBPα as well as adipose tissue-specific adiponectin, GLUT4, LPL, and FABP4—are the master regulators of adipogenesis. ATGL and HSL expression from mature adipocytes were also determined (Figure 6). The mRNA levels of the adipogenesis-associated transcription factors PPARγ and C/EBPα were significantly suppressed by 96.87% and 20%, respectively. Treatment with BAB (1 mg/mL, 2 mg/mL) decreased the expressions of adiponectin (71.3%, 89.5%) and GLUT4 (90.37%, 92.8%). The expressions of FABP4 (12.74%, 95.06%) and LPL (48.59%, 71.81%) after treatment were also strongly decreased (Figure 6A). The experiment in Figure 6B shows that mRNA levels of the lipolytic genes ATGL (303.17%, 426.79%) and HSL (456.56%, 386.04%) were higher in the treatment group than in the untreated control.

## 4. Discussion

Obesity increases the risk of metabolic disorders such as hyperglycemia, hyperlipidemia, hypercholesterolemia, and diabetes [1]. Both an increased number of adipocytes, due to enhanced differentiation of preadipocytes into adipocytes, and increased adipocyte size due to lipid accumulation are shown to participate in the expansion of adipose tissue [24]. Recent studies have found that dietary supplementation with radical scavenging compounds may lead to decreased body weight and/or several obesity-related disorders [25]. Numerous studies have suggested that oxidative stress may be the linking mechanism in the pathway leading from obesity to obesity-related diseases [26,27]. BAB exhibited the greatest antioxidant activity compared to 20 other kinds of adzuki beans (Supplementary Table S1). The IC_50_ of BAB for scavenging DPPH and ABTS radicals were 0.98 and 2.00 mg/mL, respectively (Table 1). These data demonstrated the ability of BAB to scavenge ROS, as in the hydrogen donation (DPPH assay), and to scavenge proton radicals by donating electrons (ABTS assay) [28]. Recently, Qusti *et al.* [29] demonstrated the DPPH radical scavenging activity IC_50_ < 1 mg/mL (freeze-dried weight) of various foods that were considered to have extremely high antioxidant activity. This range included red grapes (0.43 mg/mL), pomegranate (0.53 mg/mL), and black olives (0.69 mg/mL). Adzuki bean extracts are known to contain various polyphenols such as proanthocyanidins and quercetin glycosides as well as other catechins, chlorogenic acid, and genistein [10,30]. These antioxidants have been shown to inhibit proliferation and adipogenesis [1,31,32]. Also, some studies have suggested that genistein promotes lipolysis, inhibits adipogenesis in cell culture, and regulates body fat targeting the preadipocyte proliferation and differentiation [32]. Thus BAB could be useful in ameliorating pathological conditions. However, further studies to compare the activity of single and mixed antioxidant compounds from BAB are needed.

In this study, we demonstrated that BAB suppresses the differentiation of 3T3-L1 adipocytes (Figure 5). These observations were similar to those reported by Tomoko *et al.* [33], but we further elucidated the presence of BAB at different time periods during adipocyte differentiation. We found that BAB decreased lipid accumulation in 3T3-L1 cells and inhibited their differentiation into mature adipocytes, suggesting that BAB interferes with the proliferation and differentiation process, prohibiting cells from acquiring the complete adipose phenotype (Figure 1 and Figure 2). BAB treatment during Ep exerted a greater effect than treatment in Tp for inhibiting the formation of lipid droplets (Figure 3). These results suggest that inhibition of adipocyte differentiation by BAB may begin in the early stage of adipocyte differentiation. Differentiation of preadipocytes undergoes morphological and biochemical transition from growth arrest of confluent preadipocytes, re-entry into the cell cycle for an additional several rounds of postconfluent mitosis (known as MCE), and terminal differentiation into mature adipocytes followed by changes in genetic programs for lipid synthesis and storage [34,35]. Especially, MCE is reported to be necessary for the progression through subsequent steps in the differentiation program, and selective inhibition of each cell cycle step is sufficient to totally block the adipogenesis process [4]. In fact, blocking the entry of 3T3 cells into S phase at the time of MCE completely inhibits the adipose conversion program [35]. Also, inhibition of DNA synthesis in 3T3-F442A cells prevents formation of fat cells [36]. From this point of view, inhibition of DNA synthesis by BAB treatment, which we confirmed by BrdU incorporation, an S phase-specific marker, leads to inhibiting differentiation from early phase to terminal periods (Figure 2 and Figure 3). C/EBPβ is expressed in the early phase after MDI induction. Activation of C/EBPβ is clearly associated with MCE, as cells without C/EBPβ cannot complete clonal expansion [35]. At this point in the differentiation program, C/EBPβ is unable to bind DNA and thus cannot function as a transcriptional activator [4]. Thus, delayed acquisition of DNA binding function by C/EBPβ seems to forestall expression of PPARγ and C/EBPα [35]. BAB treatment blocked the mRNA expression of C/EBPβ within the early phase after induction by approximately 56%–64% compared with those untreated controls (Figure 4). These findings, together with our data showing that BAB inhibits proliferation and reduces BrdU incorporation rate and, according to priority, blocks the mRNA expression of C/EBPβ, as well as decreases lipid accumulation in early periods, suggests that BAB is involved in regulation of the early stage of adipocyte differentiation.

C/EBPβ, an adipogenesis regulator at the early stage of the adipocyte life cycle, is the likely link between MCE and transcriptional activation of PPARγ and C/EBPα [4]. Our finding elucidated that BAB-suppressed mRNA expression of PPARγ and C/EBPα is likely to be a consequence of decreased C/EBPβ in the early stage of adipocyte differentiation (Figure 6). PPARγ regulates the anabolic arm of lipid metabolism [37,38]. The PPARγ and C/EBPα are considered to be the orchestrators of adipogenesis, and their expression patterns determine adipose differentiation [39]. The downstream target genes of PPARγ and C/EBPα such as FABP4 and LPL were also downregulated by BAB treatment in differentiated 3T3-L1 cells (Figure 6). The FABP4 gene, a lipid carrier expressed during adipose differentiation, had similar expression kinetics as C/EBPα; FABP4 reaches its maximum level of expression in terminally differentiated adipocytes [4]. PPARγ activates LPL expression and the TG biosynthetic pathway. Secreted LPL hydrolyses TG, releasing free fatty acid (FFA) to be re-esterified [40]. Therefore, the above genes involved in PPARγ signaling reveal that BAB inhibits adipogenesis as well as lipogenesis by regulating fatty acid transport and adipocyte differentiation. The expressions of other genes involved in glucose uptake were also attenuated by BAB treatment, specifically adiponectin and GLUT4. The expression of GLUT4, which participates in insulin-dependent glucose uptake into adipocytes [41] and is thus indirectly involved in fatty acid synthesis, was significantly decreased. Insulin promotes FFA esterification into triglycerides through stimulation of GLUT4-mediated glucose uptake. Glucose can be converted to α-glycerol phosphate, the main source of the glycerol backbone of TG [40]. Marked expression of GLUT4 in 3T3-L1 adipocytes occurs late in the differentiation process relative to other adipocyte-specific genes [42]. C/EBPα directly regulates the expression and/or translocation of the GLUT4; the decrease in the expression of C/EBPα would directly impair the function of GLUT4, which would decrease glucose transport in fat cells [43]. Therefore, in addition to suppression of PPARγ and C/EBPα, BAB treatment was effective in downregulating adipogenic-associated genes, as mentioned above. On the other hand, HSL and ATGL, involved in triglyceride lipolysis, were upregulated by BAB. ATGL participates in triglyceride-specific hydrolysis and, hence, HSL plays an important role in lipid metabolism [44].

**Figure 7:**
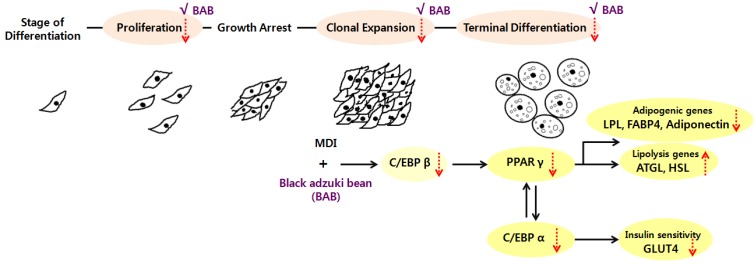
The possible mechanism of black adzuki bean-mediated inhibition of adipocyte differentiation.

Overall, these results indicate that BAB impairs the proliferation and MCE, and subsequently inhibits the adipogenesis of 3T3-L1 preadipocytes by down regulating many genes associated with lipid accumulation during differentiation of 3T3-L1 cells (Figure 7). Thus, we suggest that BAB has shown its antiadipogenic activity through modulating the differentiation phase and therefore could be a potential candidate in the prevention and attenuation of lipid accumulation in adipose tissue. However, a further study elucidating the therapeutic role of BAB *in vivo* should be performed using a diet-induced obese animal model.

## 5. Conclusions

Adipogenesis plays an important role in expansion of adipose tissue by promoting the development of preadipocytes into mature adipocytes with the accumulation of lipid droplets. Therefore, averting fat accumulation may require the inhibition of the adipogenic process along with lipolysis [27].

In summary, BAB inhibited proliferation, reduced BrdU incorporation rate, and downregulated the mRNA expression of C/EBPβ crucial for adipogenesis. Further, it decreased the lipid accumulation in early periods of adipogenesis, suggesting that BAB is involved in regulation of the early stage of adipocyte differentiation. BAB can significantly reduce the expression of C/EBPβ, which acts an important factor for initiating differentiation. Furthermore, our results showed that BAB potentially suppresses lipid accumulation and also many genes essential for lipid metabolism as well as adipogenesis. Taken together, our study provides new insights into the molecular basis underlying the antiadipogenic property of the black adzuki bean.

## Supplementary Files

Supplementary File 1
