# Supplementary material for: Effects of Black Adzuki Bean (Vigna angularis) Extract on Proliferation and Differentiation of 3T3-L1 Preadipocytesinto Mature Adipocytes
Source: Nutrients. 2015 Jan 6;7(1):277–92. doi: 10.3390/nu7010277 (PMC4303839; doi:10.3390/nu7010277)
Supplement: Supplementary File 1 [file nutrients-07-00277-s001.docx]

Supplementary Information

**Table S1.** Antioxidant activities of the adzuki bean extracts, as determined by the DPPH and ABTS^+^ assay.

| **Samples** | **Radical Scavenging Activities(IC_50_ = mg/mL)** | |
| --- | --- | --- |
|  | **DPPH** | **ABTS^+^** |
| *Positive control* | | |
| Ascorbic acid | 0.03 ± 0.00 ^c^ | - |
| Trolox | - | 0.10 ± 0.00 ^i^ |
| *Adzuki bean extract* | | |
| sample 1 (BAB) | 0.83 ± 0.03 ^c^ | 2.38 ± 0.09 ^gh^ |
| sample 2 | 1.10 ± 0.07 ^c^ | 2.42 ± 0.03 ^g^ |
| sample 3 | 1.17 ± 0.25 ^c^ | 2.52 ± 0.02 ^ef^ |
| sample 4 | 1.31 ± 0.09 ^c^ | 2.62 ± 0.02 ^cd^ |
| sample 5 | 0.94 ± 0.11 ^c^ | 2.4 ± 0.02 ^g^ |
| sample 6 | 1.49 ± 0.23 ^c^ | 2.65 ± 0.00 ^c^ |
| sample 7 | 5.51 ± 0.82 ^b^ | 2.4 ± 0.01 ^g^ |
| sample 8 | 1.38 ± 0.02 ^c^ | 2.45 ± 0.02 ^fg^ |
| sample 9 | 1.32 ± 0.22 ^c^ | 2.42 ± 0.00 ^g^ |
| sample 10 | 1.38 ± 0.04 ^c^ | 2.43 ± 0.02 ^g^ |
| sample 11 | 1.21 ± 0.25 ^c^ | 2.34 ± 0.02 ^h^ |
| sample 12 | 1.06 ± 0.05 ^c^ | 2.42 ± 0.00 ^g^ |
| sample 13 | 4.77 ± 0.23 ^b^ | 2.46 ± 0.02 ^fg^ |
| sample 14 | 15.40 ± 0.94 ^a^ | 11.66 ± 0.02 ^a^ |
| sample 15 | 1.14 ± 0.18 ^c^ | 2.44 ± 0.02 ^g^ |
| sample 16 | 5.89 ± 0.38 ^b^ | 2.74 ± 0.10 ^b^ |
| sample 17 | 1.06 ± 0.20 ^c^ | 2.43 ± 0.01 ^g^ |
| sample 18 | 5.07 ± 0.38 ^b^ | 2.68 ± 0.05 ^bc^ |
| sample 19 | 6.68 ± 0.92 ^b^ | 2.57 ± 0.03 ^de^ |
| sample 20 | 1.54 ± 0.01 ^c^ | 2.46 ± 0.01 ^fg^ |

The concentration of adzuki bean extract providing 50% inhibition (IC_50_) was calculated from the graph-plotted inhibition percentage against adzuki bean extract concentration (mg/mL). Data are expressed as means ± SD and a different letter indicates a significant difference among adzuki bean extract, according to ANOVA with Duncan’s multiple range test (*p* < 0.05). BAB: black adzuki bean.

|  |  |
| --- | --- |
| **(A)** | **(B)** |

**Figure S1.** Antioxidant activities of the bean extracts as determined by the (**A**) DPPH assay and (**B**) ABTS^+^ assay. BAB compared with RAB by Independent *t*-test (* *p* < 0.05).
BAB: black adzuki bean; RAB: red adzuki bean; BB: black soybean.

© 2015 by the authors; licensee MDPI, Basel, Switzerland. This article is an open access article distributed under the terms and conditions of the Creative Commons Attribution license (http://creativecommons.org/licenses/by/4.0/).
